# Telehealth Use in Community Health Clinics by Ethnicity and Language

**DOI:** 10.1001/jamahealthforum.2025.3336

**Published:** 2025-08-22

**Authors:** Miguel Marino, Dang Dinh, Jennifer A. Lucas, Gretchen Mertes, Nathalie Huguet, Beverly B. Green, John Heintzman

**Affiliations:** 1 Department of Family Medicine, Oregon Health & Science University, Portland; 2 Biostatistics Group, School of Public Health, Oregon Health & Science University–Portland State University, Portland; 3 OCHIN, Portland, Oregon; 4 Kaiser Permanente Washington Health Research Institute, Seattle, Washington; 5 Department of Health Systems Science, Kaiser Permanente Bernard J. Tyson School of Medicine, Pasadena, California

## Abstract

This cohort study describes video- or telephone-based clinician visits among Latino patients, with English or Spanish language preference, and non-Hispanic White patients in the US from 2019 to 2023.

## Introduction

The COVID-19 pandemic led to increases in telehealth use, with 37.0% of US adults using telehealth in 2021.^1^ While findings on telehealth use by ethnicity are mixed or limited,^1,2,3^ uncertainty remains about postpandemic patterns and differences by ethnicity and language groups. There is limited understanding of telehealth patterns in community health centers (CHCs), wherein Latino people with low income, disproportionately receive primary care.

This study describes telehealth use over time among patients in CHCs across 32 US states. Understanding telehealth use by ethnicity and language is key to identifying disparities and guiding equitable clinical and policy decisions regarding telehealth.

## Methods

We used electronic health record data from the OCHIN CHC network. We included patients (aged ≥18 years) who self-identified as Latino, with English or Spanish language preference, or non-Hispanic White with 1 or more ambulatory or telehealth (video and/or telephone) visits from April 1, 2019, to December 31, 2023 (eMethods in Supplement 1). The Oregon Health & Science University Institutional Review Board approved this cohort study and waived informed consent because deidentified data were used. We followed the STROBE reporting guideline.

Main outcomes were (1) any telehealth use vs in-person only and (2), among any telehealth users, a categorical variable denoting majority telehealth (≥50% visits via telehealth), minimal telehealth (>1 but <50% visits via telehealth), or single telehealth visit. Within each ethnicity-language stratum, we identified patient characteristics and visually described visit rates among telehealth users. Statistical analysis was performed from November 2024 to February 2025 using Stata, version 14.1 (StataCorp LLC).

## Results

Of the 2 466 404 patients (1 418 043 females [57.5%]), 32.4% identified as Latino with Spanish preference, 17.8% as Latino with English preference, and 49.8% as non-Hispanic White; 38.0% used telehealth. The proportion of telehealth visits increased during the pandemic across all groups, peaking in 2021 to 2022 before declining in 2023. Among Latino patients with English preference, telehealth use ranged from 0.1% in 2019, 16.6% in 2020, 28.8% in 2021, 28.9% in 2022, to 25.3% in 2023.

Among those 65 years or older, more Latino patients with Spanish preference used any telehealth than Latino patients with English preference and non-Hispanic White patients (46.9% vs 39.8% and 33.9%) (Table). Telehealth use was higher among patients with vs without chronic conditions (eg, hyperlipidemia and diabetes), especially among Latino patients.

**Table.** Characteristics of Patients With Any Telehealth and In-Person Only Visits by Ethnicity and Language Groups From 2019 to 2023^a^

Characteristic	Total, No. (%) (N = 2 466 404)		Patients, No. (%)					
			Latino with Spanish preference (n = 798 347)		Latino with English preference (n = 439 514)		Non-Hispanic White (n = 1 228 543)	
	Any telehealth	In-person only	Any telehealth	In-person only	Any telehealth	In-person only	Any telehealth	In-person only
Patients, No.	954 808 (38.7)	1 511 596 (61.3)	327 743 (41.1)	470 604 (58.9)	177 437 (40.4)	262 077 (59.6)	449 628 (36.6)	778 915 (63.4)
Sex								
Female	595 120 (42.0)	822 923 (58.0)	215 922 (44.9)	265 500 (55.1)	114 425 (43.8)	146 580 (56.2)	264 676 (39.2)	410 843 (60.8)
Male	359 688 (34.3)	688 673 (65.7)	111 751 (35.3)	205 104 (64.7)	62 985 (35.3)	115 497 (64.7)	184 952 (33.4)	368 072 (66.6)
Age, y								
18-29	247 245 (35.7)	444 560 (64.3)	52 699 (33.3)	105 686 (66.7)	87 609 (39.0)	137 044 (61.0)	106 937 (34.6)	201 830 (65.4)
30-39	207 745 (38.4)	332 574 (61.6)	75 354 (38.4)	120 914 (61.6)	39 572 (40.8)	57 425 (59.2)	92 819 (37.6)	154 235 (62.4)
40-49	183 719 (40.8)	266 987 (59.2)	87 308 (43.1)	115 287 (56.9)	23 262 (42.2)	31 796 (57.8)	73 149 (37.9)	119 904 (62.1)
50-64	226 471 (41.5)	319 599 (58.5)	82 190 (46.5)	94 464 (53.5)	21 876 (43.8)	28 060 (56.2)	122 405 (38.3)	197 075 (61.7)
≥65	89 628 (37.7)	147 876 (62.3)	30 192 (46.9)	34 253 (53.1)	5118 (39.8)	7752 (60.2)	54 318 (33.9)	105 871 (66.1)
Insurance status^b^								
Never insured	89 636 (18.0)	408 187 (82.0)	54 780 (20.1)	217 777 (79.9)	13 215 (17.8)	60 962 (82.2)	21 641 (14.3)	129 448 (85.7)
Some private	170 042 (32.6)	350 771 (67.4)	51 104 (41.7)	71 335 (58.3)	28 779 (33.1)	58 101 (66.9)	90 159 (28.9)	221 335 (71.1)
Some private and public	129 450 (67.0)	63 620 (33.0)	52 376 (75.4)	17 051 (24.6)	20 721 (67.5)	9993 (32.5)	56 353 (60.6)	36 576 (39.4)
Some public	565 680 (45.1)	689 018 (54.9)	169 483 (50.8)	164 441 (49.2)	114 722 (46.3)	133 021 (53.7)	281 475 (41.8)	391 556 (58.2)
Household income^c^								
Above and below 138% of FPL	164 821 (63.7)	93 989 (36.3)	62 624 (65.2)	33 366 (34.8)	28 036 (66.0)	14 467 (34.0)	74 161 (61.6)	46 156 (38.4)
Always equal to or above 138% of FPL	112 173 (30.3)	257 990 (69.7)	22 567 (30.6)	51 207 (69.4)	19 991 (31.3)	43 841 (68.7)	69 615 (29.9)	162 942 (70.1)
Always below 138% of FPL	595 216 (39.9)	896 395 (60.1)	228 314 (40.2)	339 789 (59.8)	113 780 (41.0)	163 786 (59.0)	253 122 (39.2)	392 820 (60.8)
Never documented	82 598 (23.9)	263 222 (76.1)	14 238 (23.5)	46 242 (76.5)	15 630 (28.1)	39 983 (71.9)	52 730 (23.0)	176 997 (77.0)
Ever smoke^d^								
No	816 628 (37.4)	1 364 858 (62.6)	313 799 (40.7)	456 692 (59.3)	160 660 (39.4)	247 278 (60.6)	342 169 (34.1)	660 888 (65.9)
Yes	138 180 (48.5)	146 738 (51.5)	13 944 (50.1)	13 912 (49.9)	16 777 (53.1)	14 799 (46.9)	107 459 (47.7)	118 027 (52.3)
BMI category								
No information	121 159 (23.0)	405 076 (77.0)	29 362 (22.0)	103 828 (78.0)	22 672 (25.3)	67 022 (74.7)	69 125 (22.8)	234 226 (77.2)
Underweight: <18.5	10 367 (41.6)	14 534 (58.4)	1119 (40.9)	1619 (59.1)	1559 (42.1)	2145 (57.9)	7689 (41.7)	10 770 (58.3)
Normal: 18.5-24.9	169 731 (39.8)	257 258 (60.2)	40 493 (41.0)	58 186 (59.0)	27 597 (39.8)	41 705 (60.2)	101 641 (39.2)	157 367 (60.8)
Overweight: 25-29.9	262 947 (42.0)	363 417 (58.0)	109 063 (43.7)	140 761 (56.3)	43 468 (42.5)	58 757 (57.5)	63 569 (40.3)	94 279 (59.7)
Obesity: ≥30	390 606 (45.3)	471 311 (54.7)	147 706 (47.1)	166 210 (52.9)	82 141 (47.0)	92 448 (53.0)	160 757 (43.1)	212 653 (56.9)
US region								
Midwest	114 928 (28.9)	282 956 (71.1)	24 895 (20.7)	95 214 (79.3)	17 593 (31.2)	38 743 (68.8)	72 440 (32.7)	148 999 (67.3)
Northeast	112 455 (40.3)	166 757 (59.7)	29 125 (38.4)	46 733 (61.6)	19 761 (43.4)	25 745 (56.6)	63 569 (40.3)	94 279 (59.7)
South	43 241 (16.4)	221 171 (83.6)	12 847 (13.9)	79 858 (86.1)	5729 (17.7)	26 664 (82.3)	24 665 (17.7)	114 649 (82.3)
West	684 184 (44.9)	840 712 (55.1)	260 876 (51.2)	248 799 (48.8)	134 354 (44.0)	170 925 (56.0)	288 954 (40.7)	420 988 (59.3)
Hypertension ever^d^								
No	661 009 (35.7)	1 191 972 (64.3)	219 544 (37.0)	373 819 (63.0)	141 316 (37.9)	231 534 (62.1)	300 149 (33.8)	586 619 (66.2)
Yes	293 799 (47.9)	319 624 (52.1)	108 199 (52.8)	96 785 (47.2)	36 121 (54.2)	30 543 (45.8)	149 479 (43.7)	192 296 (56.3)
Hyperlipidemia ever^d^								
No	556 837 (32.4)	1 162 820 (67.6)	159 359 (31.6)	344 996 (68.4)	123 286 (35.2)	226 729 (64.8)	274 192 (31.7)	591 095 (68.3)
Yes	397 971 (53.3)	348 776 (46.7)	168 384 (57.3)	125 608 (42.7)	54 151 (60.5)	35 348 (39.5)	175 436 (48.3)	187 820 (51.7)
Heart disease or heart failure ever^d^								
No	908 419 (38.3)	1 462 038 (61.7)	317 532 (40.7)	463 426 (59.3)	173 102 (40.1)	258 928 (59.9)	417 785 (36.1)	739 684 (63.9)
Yes	46 389 (48.3)	49 558 (51.7)	10 211 (58.7)	7178 (41.3)	4335 (57.9)	3149 (42.1)	31 843 (44.8)	39 231 (55.2)
Diabetes diagnosis ever^d^								
No	781 044 (36.7)	1 345 890 (63.3)	240 774 (37.7)	398 367 (62.3)	152 915 (38.7)	242 610 (61.3)	387 355 (35.5)	704 913 (64.5)
Yes	173 764 (51.2)	165 706 (48.8)	86 969 (54.6)	72 237 (45.4)	24 522 (55.7)	19 467 (44.3)	62 273 (45.7)	74 002 (54.3)
HbA_1c_ screening ever^d^								
No	362 060 (25.6)	1 054 789 (74.4)	74 760 (21.3)	275 504 (78.7)	73 968 (27.4)	196 285 (72.6)	213 332 (26.8)	583 000 (73.2)
Yes	592 748 (56.5)	456 807 (43.5)	252 983 (56.5)	195 100 (43.5)	104 469 (61.1)	65 792 (38.9)	236 296 (54.7)	195 915 (45.3)
Lipid screening ever^d^								
No	388 035 (27.4)	1 026 633 (72.6)	97 751 (25.5)	285 462 (74.5)	81 798 (29.3)	197 788 (70.7)	208 486 (27.7)	543 383 (72.3)
Yes	566 773 (53.9)	484 963 (46.1)	229 992 (55.4)	185 142 (44.6)	95 639 (59.8)	64 289 (40.2)	241 142 (50.6)	235 532 (49.4)

Abbreviations: BMI, body mass index (calculated as weight in kilograms divided by height in meters squared); FPL, federal poverty level; HbA_1c_, hemoglobin A_1c_.

^a^ Patients categorized into the telehealth group must have had at least 1 telehealth visit; they may have also had in-person visits. Data were obtained from the OCHIN national network of community health centers across 32 states (Alaska, Alabama, Arkansas, Arizona, California, Colorado, Connecticut, Georgia, Iowa, Idaho, Illinois, Indiana, Louisiana, Massachusetts, Maryland, Maine, Minnesota, Missouri, Montana, North Carolina, Nebraska, New Jersey, New York, Ohio, Oklahoma, Oregon, Pennsylvania, South Carolina, Tennessee, Texas, Washington, and Wisconsin).

^b^ Insurance status was assessed at each visit. “Some” indicates the patient had this insurance type at some proportion of their total visits and no other insurance type at other visits.

^c^ Household income information was collected annually; hence, income level may change over time.

^d^ An outcome was flagged when its result was not missing and greater than 0.

Among telehealth users (Figure), visit rates increased from 2019 to 2020 across all groups, with non-Hispanic White patients consistently showing the highest rates. Latino patients with Spanish preference had the lowest visit rates, which plateaued from 2021 to 2023. Following the 2020 surge, the proportion of majority telehealth visits generally declined across all groups. Non-Hispanic White patients maintained a higher proportion of majority telehealth visits from 2021 to 2023 compared with Latino groups.

**Figure.**
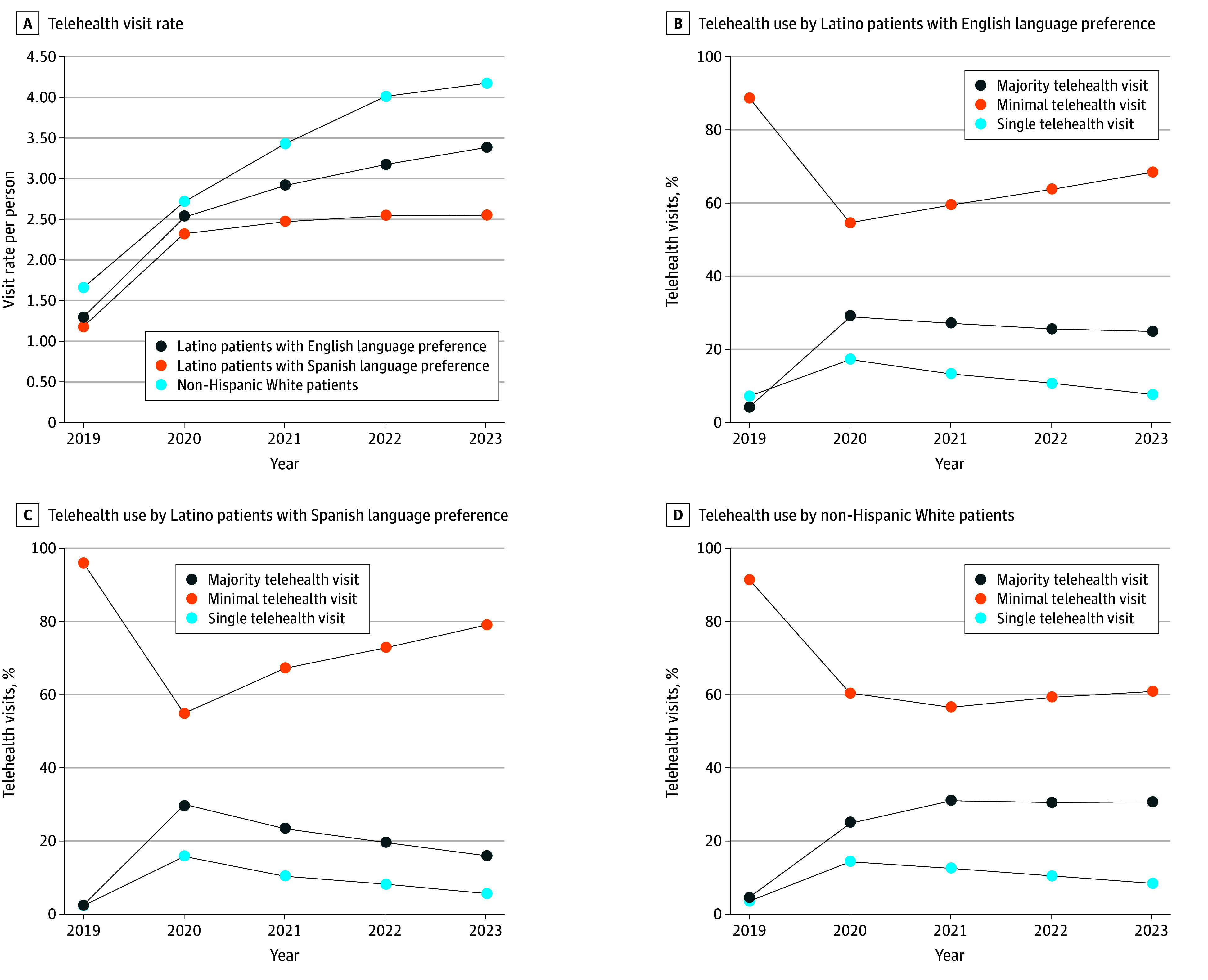
Telehealth Use and Patterns in US Community Health Centers by Ethnicity and Language From 2019 to 2023 Majority telehealth indicates 50% or more visits were via telehealth, minimal telehealth indicates more than 1 but less than 50% visits were via telehealth, and single telehealth indicates 1 visit in each of the study year.

## Discussion

Telehealth use in CHCs was similar to national averages,^1^ with lower rates among Latino patients—especially those with Spanish preference—than non-Hispanic White patients; however, this use varied by age. Lower rates may reflect adverse video-visit experiences,^3^ barriers in incorporating interpreters into telehealth visits, or limited digital literacy.^4^ Nevertheless, Latino patients’ substantial telehealth use suggest expanded access could enhance care for this large, low-income population.

In this large multistate sample, older Latino patients (aged ≥65 years) with Spanish preference demonstrated higher telehealth use than comparators, similar to other research.^1,5^ These findings challenge assumptions about age and telehealth use, suggesting value in improving access for older adults.

Study limitations include inability to distinguish visit types and limited generalizability beyond CHCs. Future research should assess telehealth’s implications for care and health outcomes for Latino patients and explore use across other racial and ethnic groups, considering language, culture, and clinical contexts.

Our findings provide timely evidence to guide health policy as the nation shifts from emergency telehealth expansion to a more sustainable framework, highlighting clinical priorities. The findings point to how evolving state and federal policies-may shape CHCs’ ability to deliver telehealth equitably.

## Notes

Supplement 1. **eMethods.** Variable Information and Missing Data Approach
eReferences

Supplement 2.
Data Sharing Statement
