# Supplementary material for: Telehealth Use in Community Health Clinics by Ethnicity and Language
Source: JAMA Health Forum. 2025 Aug 22;6(8):e253336. doi: 10.1001/jamahealthforum.2025.3336 (PMC12374211; doi:10.1001/jamahealthforum.2025.3336)
Supplement: Supplement 1. — eMethods. Variable Information and Missing Data Approach eReferences [file jamahealthforum-e253336-s001.pdf]

Supplemental Online Content

Marino M, Dinh D, Lucas JA, et al. Telehealth use in community health clinics by ethnicity and language. *JAMA Health Forum*. 2025;8(8):e253336.
doi:10.1001/jamahealthforum.2025.3336

eMethods. Variable Information and Missing Data Approach

eReferences

This supplemental material has been provided by the authors to give readers additional information about their work.

© 2025 Marino M et al. *JAMA Health Forum*

eMethods. Variable Information and Missing Data Approach

Telehealth visits

The telehealth designation was initially captured using the OCHIN variable "ENC_TYPE" which is a standard variable included in the PCORnet Common Data Model. Telehealth is defined as these visits: "Includes telemedicine or virtual visits, which can be conducted via video, phone or other means." We further required documentation of either a billing code (including CPT codes and modifiers) or a variable created by OCHIN, "telehealth mode," to confirm that it was a telehealth visit. "Telehealth mode" is a variable that was added to the EHR in March 2021. The "telehealth mode" field is required to be completed to schedule a telehealth appointment, and is modifiable by the clinician at the appointment.[1] If a telehealth visit was not coded with either a billing or telehealth mode value, it was not included.

Majority, Minority and Single Telehealth Definition

We categorized patients into three groups based on telehealth use during the study period: (1) **Majority telehealth** (>50% of visits via telehealth), (2) **Minimal telehealth** (<50% of visits via telehealth, excluding those with only one telehealth visit), and (3) **Single telehealth visit**. The 50% threshold for "majority" use was selected based on precedent from a prior study using the same EHR network[2] and reflects a meaningful cutoff for distinguishing substantial telehealth engagement.

Missing Data Approach

Because of federal reporting requirements for CHCs, race, ethnicity, insurance status, household income and other key demographic characteristics are routinely collected in the OCHIN CHC EHR data, resulting in a robust and diverse sample that spans numerous geographic and social contexts where low-income patients access primary care throughout the US. Preferred language is also well-captured, with <5% missingness. For the majority of variables (e.g., sex, language), missingness was minimal (<1%). For these variables, we performed a complete case analysis given the low potential for bias and the negligible impact on overall findings. For variables with higher rates of missingness, such as income (percent of federal poverty level) and body mass index, we addressed missing data by including separate categories for "never documented" or "no information".

eReferences

1. Larson AE, Stange KC, Heintzman J, et al. Identifying virtual care modality in electronic health record data. Learn Health Syst. Jun 2024;8(Suppl 1):e10411. doi:10.1002/lrh2.10411

2. Bailey SR, Wyte-Lake T, Lucas JA, Williams S, Cantone RE, Garvey BT, Hallock-Koppelman L, Angier H, Cohen DJ. Use of Telehealth for Opioid Use Disorder Treatment in Safety Net Primary Care Settings: A Mixed-Methods Study. Subst Use Misuse. 2023;58(9):1143-1151. doi: 10.1080/10826084.2023.2212378. Epub 2023 May 11. PMID: 37170596; PMCID: PMC10396057.

© 2025 Marino M et al. *JAMA Health Forum*
