# Supplementary material for: Telehealth Use in Community Health Clinics by Ethnicity and Language
Source: JAMA Health Forum. 2025 Aug 22;6(8):e253336. doi: 10.1001/jamahealthforum.2025.3336 (PMC12374211; doi:10.1001/jamahealthforum.2025.3336)
Supplement: Supplement 2. — Data Sharing Statement [file jamahealthforum-e253336-s002.pdf]

Data Sharing Statement

Marino. Telehealth Use in Community Health Clinics by Ethnicity and Language. *JAMA Health Forum*. Published August 22, 2025. doi:10.1001/jamahealthforum.2025.3336

Data
Data available: No

Additional Information
Explanation for why data not available: Data available upon request and appropriate DUA setup
